# Distress, anxiety, depression and unmet needs in thyroid cancer survivors: a longitudinal study

**DOI:** 10.1007/s12020-021-02786-y

**Published:** 2021-06-18

**Authors:** Margherita Dionisi-Vici, Marta Fantoni, Rossana Botto, Alice Nervo, Francesco Felicetti, Ruth Rossetto, Marco Gallo, Emanuela Arvat, Riccardo Torta, Paolo Leombruni

**Affiliations:** 1 Clinical Psychology Unit, Department of Neuroscience, University of Turin, “Città della Salute e della Scienza” Hospital of Turin, Turin, Italy; 2 IMT School for Advanced Studies, Lucca, Italy; 3 Oncological Endocrinology Unit, Department of Oncology, AOU Città della Salute e della Scienza Hospital, Turin, Italy; 4 Transition Unit for Childhood Cancer Survivors, Department of Oncology, AOU Città della Salute e della Scienza Hospital, Turin, Italy; 5 Division of Endocrinology, Diabetes and Metabolism, Department of Medical Sciences, AOU Città della Salute e della Scienza Hospital, Turin, Italy

**Keywords:** Long-term survivors, Thyroid cancer, Unmet needs, Psychological distress, Longitudinal, Anxiety

## Abstract

**Purpose:**

Despite a good prognosis, thyroid cancer (TC) survivors often report psychological distress and decreased quality of life. This longitudinal study aims to evaluate TC survivors’ levels of distress, anxiety, depression and unmet needs, checking potential life events.

**Methods:**

Distress Thermometer, Hospital Anxiety Depression Scale, Supportive Care Need Survey (short form) and Interview for Recent Life Events were administered to 73 TC survivors (T0) and 44 of them were re-tested one year later (T1). Participants were at 0–5, 5–10 or >10 years from the end of their cancer-related treatments.

**Results:**

At T0, distress, anxiety and depression mean scores were 6.4, 6.8 and 5.3, while at T1 they were 5.5, 4.8 and 5.1. Only anxiety scores decreased significantly between T0 and T1. 50.7% of patients had unmet psychological needs at T0 and 50.0% at T1. Most participants were satisfied in the communicative/ informative (T0:79.5%; T1: 77.3%) and social/health care areas (T0:74.0%; T1:75.0%). The most experienced stressful events detected concerned their working areas.

**Conclusions:**

Results confirmed that patients reported distress, anxiety and depression concerns even many years after the end of treatments. Both medical and psychological surveillance are relevant to improving TC survivors’ wellbeing.

## Introduction

The number of long-term cancer survivors is expected to increase over the next few years due to improvement in early cancer diagnosis, the progress of surgical techniques and multi-disciplinary treatment advances [1–3]. Therefore cancer, once considered as a death sentence, has become a chronic illness for a growing number of surviving patients [4, 5].

Thyroid cancer (TC) is one of the most frequent endocrine neoplasia [6, 7], and generally has a good prognosis. In the United States, the reported 5-year survival rates in patients diagnosed between 2005–2011 is about 98% [6–8]. However, periodical long-term monitoring is recommended, since 15–35% of survivors can develop a loco-regional recurrence or distant metastases, even decades later [9].

The standard treatment for TC is thyroidectomy, which further requires lifelong thyroid hormone supplementation, followed by radioiodine ablation when appropriate. Perturbations in thyroid functions often have a negative impact on patient’s daily life and may cause physical complaints like fatigue, sleep disorders, dry skin, cold intolerance, weight gain and constipation, along with neuropsychological and psychiatric disturbances such as psychomotor slowing, depression or anxiety [10–12].

Despite the relatively favorable long-term outcomes [6, 7], TC survivors have higher levels of anxious and depressive symptoms and poorer quality of life than the healthy population, and have similar levels of quality of life than that other cancer patients with worst survival, suggesting that emotional distress persists after the end of treatments [8, 9, 13].

The most frequent psychological complaints that affect TC survivors include the fear of recurrence and the necessity for additional treatments, feelings of isolation, invalidation, guilt and confusion [2, 14]. Moreover, several studies have shown that the most frequent needs in TC survivors include a strong desire to receive detailed information about medical, physical and practical issues, and support for their psychological concerns [8, 15–18]. These findings highlight the importance of identifying TC survivors’ main unmet needs to allow better and more adequate care and support. TC is often labeled as a “good cancer” [19–21] and, to encourage patients, clinicians often compare it to other “worse” cancers [14]. However, this reassuring practice does not always correspond to positive responses, as seen in patients requiring communication that assigns the right value to their cancer [9, 14, 22]. The attitude of trivializing the disease’s burden may indeed represent a barrier to providing adequate psychological supportive care, especially through survivorship [18].

Despite this evidence, there are few studies focused specifically on TC survivors’ unmet needs, and these topics should be further deepened, including longitudinal observations. Thus, we conducted a longitudinal study aimed to evaluate and monitor TC survivors’ levels of distress, anxiety and depression and their unmet needs, as well as checking for the occurrence of life events.

## Methods

### Study design and participants

The sample was recruited in the “Città della Salute e della Scienza” Hospital of Turin between May 2017 and July 2018.

The eligibility criteria were having reached the age of majority and being in a disease-free status after having received TC diagnosis and the related treatments.

The exclusion criteria were not speaking Italian fluently and/or having a diagnosis for any severe psychiatric disorder and/or having cognitive impairment precluding the capacity to provide informed consent or to complete the protocol.

Seventy-six patients were invited to participate in the research by their physicians during scheduled medical follow-up visits. One of them did not meet the inclusion criteria due to structural and biochemical persistence and, for two patients, clinical information was incomplete. So the final sample consisted of 73 TC survivors. The study counted two sessions of evaluation: the first during the regular medical follow-up examination and the second a year later. 29 (39.7%) patients did not accept the re-test. This drop-out was acknowledged and there were no significant differences in distress and unmet needs between patients who did the re-test and those who did not. Drop-out was principally imputed to practical difficulties in establishing a second appointment coinciding with their medical examination (28 patients), while one patient died between T0 and T1. As a result, 44 patients received the second evaluation.

In the first evaluation (T0)—after collecting socio-demographic and clinical information—anxiety, depression and unmet needs were assessed through a set of validated rating scales. One year later (T1), the same scales were re-proposed, adding the administration of a scale evaluating the occurrence of life events.

Written informed consent was obtained from all participants and the present research was approved by “Comitato Etico Interaziendale A.O.U. San Giovanni Battista di Torino A.O. C.T.O./Maria Adelaide di Torino”: protocol number 0073054, procedure number 255, date of approval: 04/10/15. The authors affirm that the research is in conformance with the Declaration of Helsinki.

### Measures

The Distress Thermometer (DT) [23, 24] measures patients’ general level of distress via a 10-points scale (in the shape of a thermometer) ranging from 0 (“no distress”) to 10 (“extreme distress”). DT is a screening tool commonly used in the oncological field. Patients were instructed to “cross the number that best describes how much distress [they] have been experiencing during the past week”. The second part contains a “problem list” that indicates specific sources of distress. The list consists of 5 problem domains: practical, familiar, emotional, spiritual/religious, and physical. Each domain is composed of a list of problems that may be causing patients’ distress. Patients were asked to mark “yes” or “no” for each problem-related domain. A distress score ≥4 indicates clinically relevant distress [25].

The Hospital Anxiety Depression Scale (HADS) [26] is a scale specifically designed to assess depression and anxiety in patients acceding to hospital departments. It is a 14-item questionnaire, composed of two subscales: HADS-A (anxious symptoms) and HADS-D (depressive symptoms). Patients rate each item on a four-point Likert scale ranging from 0 (“not present”) to 3 (“always present”). Higher scores indicate worse mood disturbance [10]. For each subscale, a score ≥8 suggests clinically relevant anxious/depressive symptomatology [27].

The Supportive Care Need Survey-short form (SCNS-SF 34) [28, 29] is a rating scale designed to assess the unmet needs in cancer patients. It explores five domains: psychological, communicative/information, physical condition/daily life, social care/health care, and sexuality. It is composed of 34 items with 5 possible answers: absent need, satisfied need, mild need, moderate need, and high need.

The Interview for Recent Life Events (IRLE) [30] is a scale which analyses life events occurring in the previous six months. This scale is divided into different areas: work, education, economic status, health, mourning, emigration, relationship, legal problems, family and marital problems. For each area, the independence/dependence of the events from illness and their impact on patients’ lives were investigated.

### Statistical analysis

Descriptive statistics, manova, repeated measures anova, *t*-test and chi-square test were executed. The assumptions of the tests were verified and *p*-value ≤ 0.05 was considered statistically significant. For manova, age, sex, education, profession, time since the end of the treatments, use of psychotropic drugs and psychological support were considered as potential confounders. For repeated measures anova, a dichotomous variable indicating if patients had experienced life events or not (IRLE) was inserted in the statistic as a “between-subjects” factor, checking its associations with DT and HADS. Then, its association with SCNS-SF 34 at T1 was checked with chi-square test. The other socio-demographic and clinical variables were excluded from the analysis due to unbalanced groups or because they were not variables of interest. Statistical analyses were performed using SPSS version 25 (Statistical Package for Social Sciences, Chicago, IL, USA).

## Results

### Socio-demographic and clinical characteristics of the sample

A total of 73 patients were enrolled in the study, with an average age of 56.9 years (sd = 16.3; range 18–87 years), 55 were female (75.3%), with a female to male ratio of 3.1. Most of the patients were married (*n* = 49; 67.2%) and retired (*n* = 39; 53.4%). Fourteen (19.2%) were receiving psychological support, and 15 (20.5%) were treated with psychotropic drugs (anxiolytic and/or antidepressant). At T0, only 4 patients declared a previous diagnosis of psychiatric disease, referring to mood disturbances for all of them.

Concerning the types of thyroid cancer, papillary subtype was the most frequent form (56.1%, *n* = 41), followed by mixed tumor (20.6%, *n* = 15), follicular (15.1%, *n* = 11), medullary subtypes (5.5%, *n* = 4; none of them was affected by Multiple Endocrine Neoplasia type 2, MEN2) and FT-UMP (Follicular Tumor of Uncertain Malignant Potential) (2.7%, *n* = 2). All patients underwent total thyroidectomy (only one patient suffered from hypoparathyroidism), all of them were off-therapy and received cancer diagnosis on average 10.0 years (sd = 4.63) before their enrollment in the study. The mean follow-up duration was 7.9 years (sd = 4.6).

Depending on the time passed from the end of the curative cancer-related treatments, subjects were divided into three sub-groups: 0–5 years (25 patients, 34.3%), 5–10 years (28 patients, 38.3%) and >10 years (20 patients, 27.4%) (Table 1).

**Table 1:** Socio-demographic and clinical characteristics of the sample (*N* = 73).

Characteristic	*N*^a^(%)^b^
Age	
	56.9 ± 16.3
Sex	
Male	18 (24.7)
Female	55 (75.3)
Marital status	
Single	9 (12.3)
Married	49 (67.2)
Cohabiting partner	5 (6.8)
Divorced	2 (2.7)
Widow(er)	7 (9.6)
Not-cohabiting partner	1 (1.4)
Education (years)	
	11.0 ± 4.2
Profession	
Unemployed	4 (5.5)
Employed	24 (32.9)
Housewife	5 (6.8)
Retired	39 (53.4)
Student	1 (1.4)
Caregiver^c^	
Spouse	36 (49.3)
Son/Daughter	16 (21.9)
Partner	8 (10.9)
Other relatives	4 (5.4)
No caregiver	9 (12.5)
TC histotipe	
Papillary	41 (56.1)
Mixed tumor	15 (20.6)
Follicular	11 (15.1)
Medullary	4 (5.5)
Follicular Tumor of Uncertain Malignant Potential	2 (2.7)
Time since the end of treatments	
0–5	25 (34.3)
5–10	28 (38.3)
>10 years	20 (27.4)

^a^*N*, absolute frequencies

^b^%, percentage frequencies

^c^“caregiver” is meant as someone who is close to the patient (not a healthcare professional, but a familiar or a friend) and helps him even after the end of treatments (for example accompanying patients to medical visits or giving support)

### T0. Prevalence of distress, anxiety, depression and unmet needs at enrollment

The mean score at DT was 6.4 (sd = 0.9), with 76.7% of the sample (*n* = 56) above the cut-off score. As for the DT problem list, most of the patients (67.1%, *n* = 49) reported emotional problems (depression, fear, nervousness, sadness, worry and loss of interest in their usual activities). Physical problems were reported by 24.7% (*n* = 18), practical problems by 13.7% (*n* = 10), relational problems by 20.5% (*n* = 15), while spiritual/religious concerns were experienced by 12.3% (*n* = 9) of the sample.

On the HADS, the sample’s mean scores for anxiety and depression were 6.8 (sd = 4.1) and 5.3 (sd = 3.5), respectively. Levels of anxiety above the cut-off value were reported by 46.4% of participants (*n* = 34), while levels of depression above the cut-off were reported by 30.1% of patients (*n* = 21). Sixteen patients (21.9%) had both anxiety and depression scores above the cut-off. HADS scores were not associated with the psychological support and the use of psychotropic drugs.

In SCNS-SF 34, 32 patients (43.8%) did not report unmet needs in the psychological domain, 4 patients (5.5%) reported already satisfied needs, whereas the remaining 50.7% (*n* = 37) had unmet needs. Of these, 35.0% (*n* = 13) had mild, 41.0% (*n* = 15) moderate, and 24.0% (*n* = 9) severe needs. Most patients reported satisfied needs in the communicative/information area (79.5%, *n* = 58) and in the social care/health care area (74%, *n* = 54). Only 4.1% of patients (*n* = 3) were not satisfied by the communicative/information aspects received during the treatments and only one patient (1.4%) reported unmet needs in the social care/healthcare area. The other participants had no needs in these areas. Twenty patients (27.4%) experienced unmet needs in relation to physical condition/daily life domain. Nineteen patients (26%) reported unmet needs in the sexuality area (Table 2).

**Table 2:** Comparison of DT, HADS, SCNS-SF 34 between T0 and T1.

	T0 *N* = 73 *Mean (sd*^*a*^*)*	T1 *N* = 44 *Mean (sd)*	*MANOVA*^*b*^	χ^2 c^
DT^d^	6.4 (0.9)	5.5 (3.1)	4.9*	
Practical problems	0.7 (0.9)	0.56 (0.8)	11.2**	
Relational problems	0.5 (0.6)	0.2 (0.5)	3.1	
Emotional problems	3.1 (1.7)	2.3 (1.7)	2.2	
Spiritual problems	0.1 (0.3)	0.1 (0.1)	0.0	
Physical problems	5.9 (3.7)	5.4 (3.9)	3.2	
HADS^e^				
Anxiety	6.8 (4.1)	4.8 (4.1)	11.5**	
Depression	5.3 (3.5)	5.1 (4.3)	0.7	
	*N* (*%*)^g^	*N* (*%*)^g^		
SCNS-SF 34^f^				
Psychological	37 (50.7)	22 (50.0)		3.8
Communicative/information	3 (4.1)	2 (4.5)		26.4
Sexuality	19 (26.0)	11 (25.0)		4.4
Physical condition/daily life	20 (27.4)	11 (11.0)		0.5
Social care/health care	1 (1.4)	1 (2.3)		32.0

^a^sd, standard deviation

^b^MANOVA, multivariate analysis of variance

^c^χ^2^, chi-square test

^d^DT, Distress Thermometer

^e^HADS, Hospital Anxiety and Depression Scale

^f^SCNS-SF 34, Supportive Care Needs Survey

^g^*N*(%), participants with unmet needs

*p ≤ 0.05; **p ≤ 0.01

The intra-group effect is mediated by occupation for DT and HADS-A and by time since the end of treatments for practical problems (see Results section)

DT, HADS and SCNS-SF34 scores were not associated with the years which had passed from the end of the treatments, except for the “communicative/informative” area of SCNS-SF 34 (χ^2^ = 9.6, *p* ≤ 0.05), which showed different percentages among the three sub-groups. In detail, 32.0% (*n* = 8) of subjects of the 0–5 years group had no needs, compared to 7.1% (*n* = 2) in the 5–10 years, and 10% (*n* = 2) in the >10 years group. Furthermore, 60.0% (*n* = 15) of the 0–5 years group reported satisfied needs, while this percentage increased to 92.9% (*n* = 26) in the 5–10 years group, and to 85.0% (*n* = 17) in the >10 years group. (Table 3).

**Table 3:** Association between SCNS-SF34 and the duration from the end of treatments.

SCNS-SF34^a^	0–5 years	5–10 years	>10 years	Total
Communicative/informative area	*N*^*b*^*(%)*^*c*^	*N (%)*	*N (%)*	*N (%)*
Absent needs	8 (32.0)	2 (7.1)	2 (10.0)	12 (16.4)
Satisfied needs	15 (60.0)	26 (92.9)	17 (85.0)	58 (79.5)
Unmet needs	2 (8.0)	0 (0.0)	1 (5.0)	3 (4.1)
Psychological area				
Absent needs	13 (52.0)	14 (50.0)	5 (25.0)	32 (43.8)
Satisfied needs	1 (4.0)	3 (10.7)	0 (0.0)	4 (5.5)
Unmet needs	11 (44.0)	11 (39.3)	15 (75.0)	37 (50.7)
Physical condition/daily life				
Absent needs	17 (68.0)	20 (71.4)	11 (55.0)	48 (65.8)
Satisfied needs	3 (12.0)	2 (7.1)	0 (0.0)	5 (6.8)
Unmet needs	5 (20.0)	6 (21.4)	9 (45.0)	20 (27.4)
Social care/health care				
Absent needs	10 (40.0)	5 (17.9)	3 (15.0)	18 (24.7)
Satisfied needs	14 (56.0)	23 (82.1)	17 (85.0)	54 (74.0)
Unmet needs	1 (4.0)	0 (0.0)	0 (0.0)	1 (1.4)
Sexuality				
Absent needs	17 (70.8)	22 (78.6)	11 (57.9)	50 (70.4)
Satisfied needs	0 (0.0)	1 (3.6)	1 (3.6)	2 (2.8)
Unmet needs	7 (29.2)	5 (17.9)	7 (36.8)	19 (26.8)

Only communicative/informative area needs were significantly associated with the duration from the end of treatments (χ^2^ = 9.7, *p* ≤ 0.05)

^a^SCNS-SF 34, Supportive Care Needs Survey

^b^*N*, absolute frequencies

^c^%, percentage frequencies

### T1. Participants’ distress, anxiety, depression and unmet needs after one year

One year after the first evaluation the average score on the DT scale was 5.5 (sd = 3.1), and 81.8% of participants (*n* = 34) were above the cut-off.

On HADS, the average score for anxiety decreased to 4.8 (sd = 4.1), with 10 (22.7%) patients above the cut-off. The average score for depression remained almost unchanged at 5.1 (sd = 4.3), with 10 patients (22.7%) above the cut-off. Twelve patients (27.3%) were above the cut-off for both anxiety and depression. No significant differences emerged in HADS mean scores between patients who had completed T0 and T1 evaluations and patients who had completed only T0 evaluation.

In SCNS-SF34 scores, 20 patients (45.5%) did not report unmet needs related to psychological domain and 2 patients (4.5%) were already satisfied. Twenty-two patients (50.0%) still had unmet needs in the psychological area, with 8 (36.4%) reporting mild, 9 (40.9%) moderate, and 5 (22.7%) severe needs. The communicative/ informative area reported satisfied needs by almost the entire sample (*n* = 34, 77.3%) with only 2 patients (4.5%) still presenting unmet needs, while 8 patients (18.2%) did not feel this need. Similarly, 33 patients (75.0%) had satisfied needs in the social care/health care domain, with only 1 patient (2.3%) still unsatisfied; 10 participants (22.7%) did not report needs among this area. Regarding the physical/everyday life domain, 11 patients (25.0%) reported unmet needs, and 11 participants (25.0%) reported unmet needs related to the sexuality area (Table 2). No significant differences emerged in SCNS-SF34 scores between patients who completed T0 and T1 evaluations and patients who completed only the T0 evaluation.

Regarding the occurrence of life events between T0 and T1, the most experienced stressing events concerned the working area (*n* = 24; 54.5%), the family area (*n* = 11; 25.0%), the mourning area (*n* = 10; 22.7%) and the health area (*n* = 10, 22.7%).

As for the working area, 17 patients out of 24 (70.8%) reported a negative impact on their lives, and 5 (20.8%) considered the event as likely/almost certainly resulting from the illness.

As for the familial area, 6 patients out of 11 (54.5%) reported a negative impact on daily life, while 2 (18.2%) felt these events as closely related to the illness. As for the mourning area, 9 patients out of 10 (90.0%) reported a negative impact on daily life with no relation to their illness. As for the health area, 8 patients out of 10 (80.0%) reported a negative impact on their lives and 2 (20,0%) considered the event related to the illness.

Comparing DT, HADS and SCNS-SF34 scores between T0 and T1, the DT mean score significantly decreased between T0 and T1 (F = 4.9, *p* ≤ 0.01*)*. The intra-group effect was mediated by occupation: the mean score did not fall for retired patients. Relational, emotional, spiritual and physical problems did not change from T0 to T1, whereas practical problems significantly decreased between T0 and T1 with a mediation effect of the time since the end of the treatments: the reduction was higher for patients between 5 and 10 years since the end of the treatments (0.6 vs 0.2).

The HADS anxiety mean score was significantly different between T0 and T1 (F = 11.5; *p* ≤ 0.01), with a decrease of the average anxiety scores. This result was mediated by occupation: in retired patients the HADS anxiety mean score increased for retired patients (+.58 vs −2), whereas, HADS depression mean score did not change significantly over time. At the SCNS-SF34, no significant differences emerged in unmet needs between T0 and T1 (Table 2).

Finally, no associations were found between the DT, HADS and SCNS-SF34 scores and experienced life events.

## Discussion

Data suggested that distress, anxiety and depression can affect a relevant percentage of TC survivors, even many years after the end of TC treatments. In fact, almost half of the participants reported emotional problems and unmet needs related to the psychological domain, even in different phases of follow-up.

Levels of distress, depression and psychological unmet needs assessed in the sample are similar to those evidenced in other studies on TC patients [10, 11, 25, 31–34]. Moreover, these values did not differ in relation to the lengths of time from the end of treatments and did not change along with the longitudinal evaluation, with the exception of anxiety, which decreased over time. They were not associated with the occurrence of life events.

Thus, it is conceivable that the reported psychological distress may be primarily due to patients’ condition of survivorship. The experience of a cancer diagnosis and related treatments, the fear of recurrence, the scheduled follow-up surveillance, the feeling of isolation and of not being understood may negatively impact on patients’ quality of life and psychological status [13]. In addition, it seems that the condition of being retired could impact on the change of anxiety and distress between T0 and T1, as if this condition could reduce the effect of time passing.

Considering the other domains of unmet needs, it is interesting to note that most participants were already satisfied in the communicative/information as well as in the social care/health care areas. This contrasts with most previous studies that widely described the specific need for information, communication, social and emotional support offered by physicians to TC survivors [2, 8, 15, 16, 35–39]. It is presumable that our sample was provided with exhaustive information on illness and on the related medical and practical issues by the healthcare professionals operating in the hospital where the participants were recruited. They continued to guarantee satisfactory support to patients even after the end of the treatments, promoting a good patient-physician relationship and reducing patients’ requests for care and assistance.

Remarkably, patients with a shorter follow-up and closer to TC treatments were less satisfied by communicative/informative aspects than long-term survivors. This can be due to the proximity of illness, the fear of recurrence of TC, and possibly to an inadequately consolidated relationship of trust with healthcare providers. In addition, patients closer to the end of treatments have to cope with the new condition of survivorship. This new challenge can lead them to be more demanding of healthcare providers. Over time, familiarization and adjustment with the situation make them confident with the survivorship condition, so that unmet needs tend to decrease.

Even if our study did not provide a control group, based on the literature which used a healthy control group [40, 41], it is possible to speculate that the levels of anxiety, depression, unmet needs, and QoL we found can be related to the condition of TC survivorship. In this regard, the results of the study, similarly to those emerged in other researches [8, 9, 13], seem to suggest that patients’ outcome could be explained with aspects of quality of life relative to the survivorship that are unrelated to the prognosis per se.

Our findings suggest that increasing healthcare professionals’ awareness of the psychological long-term consequences caused by TC could be relevant for clinical practice, to provide more effective supportive care, and to prevent emotional distress in survivors. In their communication with patients, clinicians should on one hand reassure patients about the generally good prognosis of the illness, but they should also attribute correct value and consideration to the psycho-social burden of TC. Good communication, comprehension and provision of adequate information are necessary to establish an optimal relationship between healthcare professionals and patients and to favor patients’ adherence to the care process. Furthermore, our results suggest that the first years after the end of treatments are crucial to constructing a solid clinician-patient alliance, which tends to stabilize through the course of the survivorship. Finally, it is also important to acknowledge the peculiar unmet needs of TC survivors, to focus interventional efforts on targeted critical issues, such as the psychological area.

The application of the Interview for Recent Life Events is a strength of this study. This test allowed control of a huge list of possible life events that could have an impact on the follow-up evaluation.

Another point of strength concerns the longitudinal design of the study, which allowed us to monitor sample changes over time. Also, the patients were divided into three groups according to the number of years since the end of the treatments. These groups were well balanced, offering us the possibility to check the periods since the treatments.

The study also has some limitations. The first are the small size of the examined cohort and the absence of a control group. Moreover, the drop-out encountered during T1 should be discussed. 39.7% of patients were not evaluated for the re-test but principally for practical reasons. Furthermore, there were no differences in distress and unmet needs between patients who did the re-test and those who did not. Thus, we can hypothesize that drop-out has a limited impact on our results. Another limitation of the study is not having considered the psychological status and unmet needs according to the TC histotype. Particularly, medullary TC has a higher clinical impact and a worse prognosis compared with differentiated TC. Since in our sample, there were only four and none had MEN2, we were unable to perform subgroup analysis for these patients. Moreover, although there were not association between the patients’ symptoms and previous psychic comorbidity, the use of psychotropic drugs and the psychological support, we do not have detailed information on previous psychiatric disorders or symptoms before TC (i.e., the time of the diagnosis and the duration), and on the use of psychotropic drugs and the psychological support (i.e., the start and the duration). Future research should deepen the aspects relative to the psychic comorbidity and its treatment.

Finally, a longer observational period (>1 year) would have provided a better understanding of changes of patients’ psychological status.

## Conclusions

The study confirms an emotional burden among TC survivors. Distress, anxiety, depression, and psychological concerns are still reported even many years after the end of treatments, as is confirmed through longitudinal observation.

A good relationship and alliance between patients and healthcare providers, based on communication, trust and support, is relevant for long-term survivorship with as few unmet needs as possible.

Finally, medical as well as psychological surveillance and support are important to improve TC survivors’ wellbeing.

## Data Availability

Data and material are available from the authors upon request.
